# Imageless Navigation Improves Intraoperative Monitoring of Leg Length Changes during Total Hip Arthroplasty for Legg-Calve-Perthes Disease: Two Case Reports

**DOI:** 10.1155/2018/4362367

**Published:** 2018-07-09

**Authors:** Ritesh R. Shah, Varsha Gobin, Jeffrey M. Muir

**Affiliations:** ^1^Department of Orthopedic Surgery, Illinois Bone & Joint Institute, Morton Grove, IL, USA; ^2^Department of Orthopedic Surgery, Advocate Lutheran General Hospital, Park Ridge, IL, USA; ^3^Department of Orthopedic Surgery, NorthShore University HealthSystem-Skokie Hospital, Skokie, IL, USA; ^4^Department of Psychology, Faculty of Science, University of Waterloo, Waterloo, ON, Canada; ^5^Intellijoint Surgical, Waterloo, ON, Canada

## Abstract

Legg-Calve-Perthes disease is a rare condition characterized by avascular necrosis and malformation of the femoral head. For many patients, total hip arthroplasty (THA) is the only viable treatment option; however, there are challenges associated with THA in this population, primarily the equalization of leg lengths. Here, we present two cases of Legg-Calve-Perthes disease treated via total hip arthroplasty with the assistance of an imageless, computer-assisted navigation device. In each case, the device provided intraoperative data on leg length in real time, allowing for improved accuracy of component placement. Postoperative leg lengths were confirmed to be equalized in each case using radiographs. These cases are, to our knowledge, the first such cases using imageless navigation during THA and demonstrate the benefits of such assistive technologies in challenging cases such as Legg-Calve-Perthes disease.

## 1. Introduction

Legg-Calve-Perthes disease (LCPD) is a relatively rare childhood disorder, occurring in between 0.4/100,000 and 29.0/100,000 children per year under age 15 years [1]. Often idiopathic in onset, LCPD is characterized by deformation and flattening of the femoral head due to avascular necrosis [2, 3], with patients experiencing symptoms ranging from leg length discrepancy (LLD) to premature osteoarthritis [2, 4–6]. Although initial treatment plans are numerous and include both conservative and surgical methods, the rate of success of such treatment is low and in many cases, effective pain relief remains elusive [2, 6].

The long-term prognosis for LCPD is poor and often includes a diagnosis of secondary hip osteoarthritis and significant leg length discrepancies which in turn result in a multitude of biomechanical complications including low back pain, knee and groin pain, and compensatory contralateral hip pain [6]. In many patients, total hip arthroplasty (THA) is the only viable treatment option; however, THA for LCPD is the subject of much debate, with some authors suggesting that LCPD patients undergoing THA bear higher risks than other THA patients and that the likelihood of unsatisfactory results following THA is higher in LCPD patients [6, 7]. Increased rates of revision surgery and a higher frequency of femur fractures and motor nerve palsies due to the technical challenges associated with THA in this population are the primary concerns for LCPD patients; however, equally challenging for surgeons is the equalization of leg lengths [6, 8], the failure of which is the most common reason for litigation against orthopedic surgeons [9]. As such, there is a need for improvements in patient-related outcomes following THA in this population.

Computer-assisted navigation is an increasingly common adjunct to THA and has demonstrated the ability to improve the accuracy of leg lengthening [10, 11]. There are, however, few studies reporting the use of this technology in THA in patients with LCPD. Here, we report 2 cases of THA for LCPD performed with the assistance of an imageless computer navigation system.

## 2. Case Presentation

### 2.1. Case 1

A 38-year old male presented with a chief complaint of chronic and worsening left hip pain. The patient reported a lifelong history of pain and disability and described his current pain as sharp, aching, burning, severe, and constant, occurring daily. Relevant history included a diagnosis of hip dysplasia at age 1 year and confirmation of Legg-Calve-Perthes disease at age 18. The patient walked with a significant limp and reported difficulties with activities of daily living such as putting on socks and shoes and pain on getting out of bed. He also reported the use of a heel lift to equalize his leg lengths and expressed frustration at his daily pain. He had attempted to manage his pain with home exercise, activity modification, strengthening, anti-inflammatories, ice, and rest at length, all without success.

On physical examination, the left leg was significantly shorter, with the leg length discrepancy measured grossly at approximately 2.5–3.0 cm. Range of motion (ROM) of left hip was decreased and measured as follows: 0–85° of flexion, 5° of internal rotation in flexion (IRF), 5° of external rotation in flexion (ERF), 30° of abduction, and 5° of adduction. Abductor strength was 4/5 bilaterally. Neurological examination was unremarkable.

Radiographs revealed left hip dysplasia with upsloping of the acetabular socket. LCPD affecting the femoral head was confirmed, with an incompletely formed femoral head noted and a leg length discrepancy measured at 2.5 cm.

The preoperative plan included left hip total hip arthroplasty. Imageless, computer-assisted navigation was used during surgery to assist with leg length equalization and component placement (off-label use).

**Figure 1:**
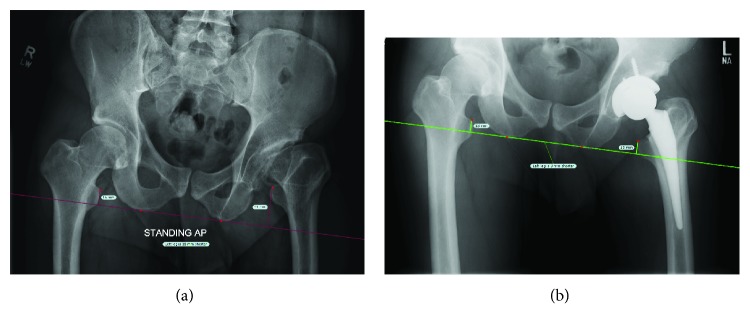
Pre- (a) and postoperative (b) radiographs for Case 1. Preoperatively, the LLD was estimated at 2.5 cm. Leg lengths were equalized postoperatively.

Surgery was successful and at three weeks postprocedure, the patient was progressing well, had experienced significant pain relief, and was satisfied with his surgery. Left hip range of motion was improved, most notably in flexion and ERF. Postoperative ROM was measured at 0–95° flexion, 5° IRF, 30° ERF, 30° abduction, and 10° adduction. Radiographs revealed that the left hip was well aligned and concentrically reduced. The left leg was lengthened by 2.5 cm, and leg lengths were even (Figure 1).

### 2.2. Case 2

A 47-year old female presented with a chief complaint of severe right-sided hip pain that was chronic in nature. Relevant history included right hip surgery at 10 years of age to address symptoms of Legg-Calve-Perthes disease. She reported no relief from this procedure and in the interim, had sought relief through multiple conservative treatments without success. The patient also reported chronic low back pain, contralateral knee pain, and right-sided groin pain and thigh pain which she attributed to a significant LLD.

On physical examination, the right leg was significantly shorter, with a noticeable LLD present. Range of motion of right hip was decreased and measured as follows: 0–80° flexion with significant pain at end range, 5° IRF with significant pain, 5° ERF, 20° abduction, and 10° adduction. Abductor strength was 4/5 bilaterally. On orthopedic testing, anterior impingement test, Patrick-FABERE, and lateral impingement tests were all positive on the right. Neurological examination was unremarkable.

Radiographic examination revealed a 3.5 cm LLD with the right leg shortened, ovoid femoral head, joint space narrowing, sclerosis, osteophytes, acetabular dysplasia, shortened femoral neck, and trochanteric overgrowth. Diagnoses of Legg-Calve-Perthes disease and secondary osteoarthritis were confirmed.

The preoperative plan included a right hip total hip arthroplasty. During surgery, computer-assisted navigation was again used to assist with component placement and monitoring of changes in leg length (off-label use).

**Figure 2:**
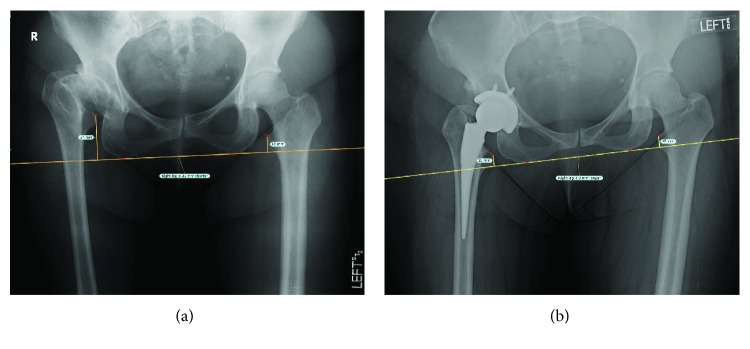
Pre- (a) and postoperative (b) radiographs of Case 2. Preoperative LLD was measured at approximately 3.5 cm. Postoperatively, leg lengths were equalized.

Surgery was successful and at three weeks postprocedure, the patient reported significant pain relief and was satisfied with the outcome of her surgery. She reported the use of a cane when walking long distances but was otherwise ambulating without the use of assistive devices and was progressing well in physical therapy. Range of motion had improved, most significantly in flexion, ERF, and abduction, and was measured at 0–95° flexion, 5° IRF, 30° ERF, 35° abduction, and 5° adduction. Radiographs revealed equalized leg lengths and implants that were well aligned and concentrically reduced (Figure 2).

## 3. Discussion

Legg-Calve-Perthes disease is a challenging condition for physicians, as conservative treatments and early surgical interventions are largely temporary and THA is often required in the long term. THA in this population, however, can be technically difficult and complex, with the potential for complications such as femoral fracture and motor nerve palsies [4–6]. Moreover, previous childhood surgeries related to LCPD often alter proximal femoral anatomy and may complicate component implantation during THA [8, 12]. Given the additional risks and complications associated with THA in patients with LCPD, there is a potential role for assistive technologies in this surgical population.

The cases presented in this report utilized an imageless, computer-assisted navigation system (Intellijoint HIP®, Intellijoint Surgical Inc., Waterloo, ON) to provide real-time intraoperative data regarding acetabular cup position and changes in leg length, data that was integral in equalizing leg lengths during surgery. To our knowledge, this marks the first such use of imageless navigation in LCPD-related THA. In both cases, a large preoperative LLD was present. Postoperatively, radiographic analysis confirmed that leg lengths were equalized. The ability of the navigation device to provide accurate intraoperative data was key in ensuring equal leg lengths postoperatively. The navigation device allowed for real-time feedback and increased the ability of the surgeon to accurately place components, thus decreasing the likelihood of unsatisfactory postoperative results. Given the prevalence of LLD in LCPD and the impact of its associated sequelae on patients' functional abilities, the ability to equalize leg lengths postoperatively is paramount. Furthermore, if LLD is not corrected accurately during primary THA, the resulting pain and functional impairment may require costly revision surgery to equalize leg lengths [13].

The biomechanical complications of LLD in LCPD are compounded by potentially significant complications such as growth plate disruption, which can further exacerbate leg shortening and increase the likelihood of a chronic limp [14]. Additionally, the high rate of neurological complications associated with THA in this patient population is also a concern [12], with such complications thought to be related to inadequate soft tissue release or excessive limb lengthening due to poor preoperative planning [12]. The use of assistive technologies may help to mitigate these concerns, as it provides feedback in real time, allowing the surgeon to alter preoperative plans based on intraoperative data. Finally, the device used in the presented cases is a new technology, one that is portable and does not require large equipment or operating room alterations, making the technology cost-effective [10]. In the current climate of increasing healthcare costs, cost-effectiveness and the potential to save costs by decreasing the likelihood of revision surgery are important factors in surgeon and facility decision-making.

## 4. Conclusion

This report summarizes two cases of Legg-Calve-Perthes disease treated with the assistance of an imageless, computer-assisted navigation device. The device provided intraoperative measurements of leg length which allowed improved accuracy with component placement and improved outcomes following surgery but limiting the likelihood of a postoperative leg length discrepancy.
